# Simplicity: The Ultimate Sophistication in Managing Type 2 Diabetes With Severe Hyperglycemia

**DOI:** 10.1111/1753-0407.70042

**Published:** 2024-12-20

**Authors:** Liehua Liu, Yanbing Li

**Affiliations:** ^1^ Department of Endocrinology The First Affiliated Hospital of Sun Yat‐Sen University Guangzhou Guangdong Province China

Diabetes mellitus, affecting 537 million people globally [1], is a chronic metabolic disorder with well‐documented “legacy effects” of early hyperglycemia on complications that persist for decades [2]. The importance of early intensive glycemic control is evidenced by long‐term follow‐up data from the United Kingdom Prospective Diabetes Study, demonstrating that it lowers the risks of mortality and both microvascular and macrovascular complications, yielding life‐long benefits [3].

Nevertheless, glycemic control in type 2 diabetes (T2DM) remains inadequate globally, particularly in China, where only fewer than 50% of patients achieve HbA1c < 7% [4]. Moreover, severe hyperglycemia at diagnosis is prevalent. A retrospective study in China showed that 44.5% of newly diagnosed patients present with HbA1c > 9% [5]. This fact poses a substantial challenge for achieving early and sustained glycemic control.

The traditional stepwise escalation approach, which gradually intensifies treatment in response to worsening hyperglycemia, often fails to achieve optimal glycemic control and prevent progressive β‐cell dysfunction. Timely initiation of combination therapy is recommended for marked hyperglycemia. Current guidelines from the American Diabetes Association recommend combination therapy when HbA1c exceeds treatment targets by 1.5% (≥ 8.5%) and insulin for HbA1c > 10%, expecting simplifying treatment after correction of glucotoxicity [6]. However, because high‐quality evidence is lacking, no standardized guidance could be provided. Indeed, more potent combination regimens may result in superior glycemic control. As shown in the EDICT study, a triple therapy regimen consisting of metformin, pioglitazone, and exenatide achieved HbA1c < 6.5% in 78% of participants over 3 years [7]. However, real‐world challenges, including adherence, tolerability, and cost, limit broader implementation of combination therapy, particularly that with injectable agents [8].

Simplified treatment regimens have consistently been an urgent need for healthcare providers and patients. Nevertheless, as Leonardo da Vinci famously said, “Simplicity is the ultimate sophistication.” Achieving both optimal glycemic control and treatment simplification requires a shift in strategy to pay more attention to the reversibility of β‐cell dysfunction in early T2DM. This requires addressing the major mechanisms of disease progression. In patients with significant hyperglycemia, glucotoxicity plays a central role in β‐cell dysfunction, reducing β‐cell secretory capacity through mechanisms such as triggering β‐cell dedifferentiation and endoplasmic reticulum stress [9, 10]. Our previous studies have shown that short‐term intensive insulin therapy (SIIT) effectively alleviates glucotoxicity, significantly improving β‐cell function and insulin sensitivity. Moreover, this approach has been shown to induce diabetes remission lasting over 1 year in more than 50% of patients [11, 12, 13, 14].

However, maintaining the benefits of SIIT remains a challenge in the long term. Factors such as the difficulty of adhering to lifestyle interventions and the impact of non‐modifiable factors like aging contribute to this complexity. The remission rate after SIIT declines over time, from 70% immediately post‐therapy to approximately 50% by the end of the first year and around 40% by the end of the second year [14]. These observations provide two key insights: First, reversing hyperglycemia and β‐cell dysfunction by SIIT may establish a robust foundation for long‐term glycemic control. Second, implementing sustained glucose‐lowering strategies after intensive therapy is crucial to prevent recurrent hyperglycemia and to protect β‐cell function over time.

Building on these understandings, we developed an “Intense‐Simplified” strategy. This approach initially employs SIIT to eliminate glucotoxicity to the maximum extent, followed by a simplified oral hypoglycemic regimen to maintain glycemic control. To evaluate the efficacy of this strategy, we conducted an open‐label, multicenter, randomized controlled trial across 15 hospitals in China [15].

The study enrolled 412 drug naïve patients newly diagnosed with T2DM whose glycated HbA1c levels exceeded 8.5%. All participants initially underwent SIIT aimed at achieving normoglycemia (fasting/pre‐meal glucose < 6.1 mmol/L, 2‐h postprandial glucose < 8.0 mmol/L) for 2 weeks to alleviate glucotoxicity comprehensively. After completing the “intense” phase, participants were randomized in a 1:1:1:1 ratio into four maintenance therapy groups and followed for 48 weeks:

1. *Linagliptin plus Metformin group (LIN + MET)*: linagliptin 5 mg/day combined with metformin 1000 mg/day.
2. *Linagliptin group (LIN)*: linagliptin 5 mg/day monotherapy.
3. *Metformin group (MET)*: metformin 1000 mg/day monotherapy.
4. *Control group*: lifestyle intervention without glucose‐lowering medications.

The primary endpoint was the proportion of participants achieving HbA1c < 7% at the end of the 48‐week follow‐up period.

Participants had an average age of 46.8 years and a mean body mass index (BMI) of 25.8 kg/m^2^. Baseline HbA1c was 11.0% ± 1.9%, with mean fasting plasma glucose (FPG) of 11.4 mmol/L and 2‐h postprandial glucose (PPG) of 20.5 mmol/L. Notably, 76% of participants presented with symptoms of severe hyperglycemia, such as polydipsia, polyuria, and weight loss.

During the “Intense” phase, SIIT effectively reduced mean FPG to 5.9 mmol/L and PPG to 13.8 mmol/L. In the subsequent “Simplified” phase, the LIN + MET group had the highest proportion of participants with HbA1c < 7% (80%), significantly higher than the control group (60%, *p* = 0.003). In the per‐protocol set (PPS), this proportion was even higher in the LIN + MET group (85.9%). The LIN and MET groups achieved HbA1c < 7% rates of 72% and 73%, respectively.

For the more stringent target of HbA1c < 6.5%, the proportions in the LIN + MET, LIN, and MET groups were 70%, 68%, and 68%, respectively, compared with 48% in the control group (*p* = 0.005). The LIN + MET group showed the greatest reduction in HbA1c from baseline, with a mean decrease of 4.8%, resulting in a final mean HbA1c of 6.2%, which was significantly lower than the other groups (LIN: 6.5%, MET: 6.4%, control: 6.7%, *p* = 0.004). This group also demonstrated the most substantial reduction in FPG, achieving an average of 6.0 mmol/L at the end of the study, which remained stable throughout the treatment period (Figure 1A–D).

**FIGURE 1:**
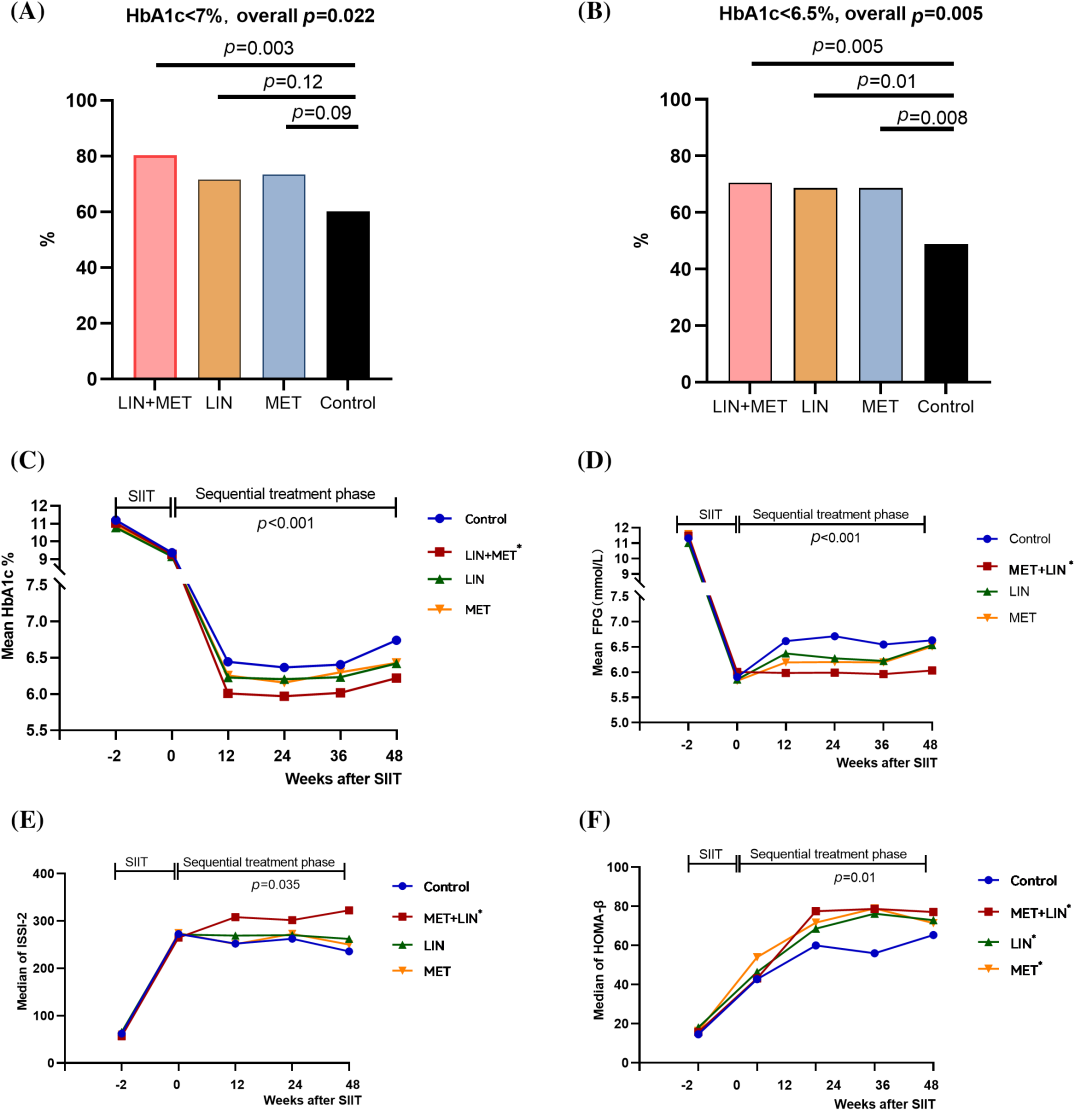
Treatment effects of the Intense‐Simplified strategy in patients with newly diagnosed type 2 diabetes with severe hyperglycemia (Adapt from ref. [15], BMJ. 2024; 387: e080122). The proportions of participants achieving HbA1c < 7.0% and HbA1c < 6.5% at week 48 are shown in A and B; Changes of HbA1c and fasting plasma glucose overtime are shown in C and D; Changes of insulin secretion sensitivity index‐2 and HOMA B are shown in E and F. SIIT, short‐term intensive insulin therapy; LIN + MET, the linagliptin+metformin group; LIN, the linagliptin group; MET, the metformin group. *, *p* < 0.0167 compared with the control group.

The remarkable glycemic control observed with the Intense‐Simplified strategy is attributed to its disease‐modifying property. The LIN + MET group exhibited continuous improvement in the Insulin Secretion‐Sensitivity Index‐2 throughout the study, significantly superior to the control group. Additionally, the subsequent intervention groups (LIN, MET, LIN + MET) demonstrated significantly higher HOMA‐B levels than the control group, highlighting the strategy's capacity to preserve β‐cell function and extend the benefits of SIIT (Figure 1E,F). Patient compliance with subsequent treatments was excellent, exceeding 85% in all three pharmacological groups. This high adherence was partly due to the simplicity of the regimens (lower doses of metformin and the favorable safety profile of DPP‐4 inhibitors). Gastrointestinal adverse events were primarily reported in the MET and LIN + MET groups, but most patients tolerated these events well, with a drug discontinuation rate below 5%.

The outcomes of the Intense‐Simplified model are strikingly impressive and significantly superior to those reported in other studies employing similar oral regimens. For example, in the well‐known VERIFY study, early combination treatment with vildagliptin and metformin reduced treatment failure rates in newly diagnosed T2DM patients (mean HbA1c 6.5%–7.5%), yet 43.6% of participants in the combination therapy group still experienced treatment failure [16]. A post hoc analysis of two randomized controlled trials found that linagliptin combined with metformin (1000 mg/day, comparable to this study) reduced HbA1c by 1.67% in early T2DM patients with moderate hyperglycemia (mean baseline HbA1c 8.7%), achieving HbA1c < 7.0% in only 51.4% and HbA1c < 6.5% in just 40.1% of patients [17]. This apparent disparity highlights the unique disease‐modifying properties of the Intense‐Simplified approach.

The Intense and Simplified modules are fundamentally integrated. The Intense module prioritizes achieving near‐normoglycemia to completely eliminate glucotoxicity, which is critical for β‐cell recovery. Evidence suggests that tighter glycemic control during SIIT—indicated by lower mean blood glucose [18] and higher time in range (TIR) [19]—strongly predicts β‐cell restoration and long‐term glycemic outcomes. The Simplified module focuses on maintaining the benefits of reversal therapy with cost‐effective, practical regimens that are easy to implement. In real‐life scenarios, glycemic fluctuations are inevitable. Basic research has demonstrated that even mild hyperglycemia can trigger β‐cell dysfunction, phenotypic changes, and dedifferentiation, leading to reduced functional β‐cell mass and worsening hyperglycemia [20]. This vicious cycle is a major challenge in long‐term management. Sequential oral therapies could minimize these fluctuations and prevent the β‐cell deterioration that might otherwise undermine the outcomes of intensive treatment.

The Intense‐Simplified strategy is not a rigid, fixed regimen but a flexible approach that could be tailored to clinical needs. The primary objective of the Intense phase is to eliminate glucotoxicity comprehensively, allowing flexibility in treatment approaches. For instance, in an outpatient setting, multiple daily injections or a regimen combining long‐acting insulin with oral hypoglycemic agents under proper supervision could be considered, provided the approaches could effectively normalize blood glucose levels. In the Simplified phase, convenient fixed‐dose combinations or regimens with stronger glucose‐lowering effects could both be considered. A frequently debated question is whether the Simplified module should prioritize glucose‐lowering agents with cardiovascular benefits, such as GLP‐1 receptor agonists (GLP‐1RA) or SGLT‐2 inhibitors (SGLT‐2i) [6]. It should be noted that sustained glycemic control in the early stages of T2DM per se could effectively reduce both microvascular and macrovascular complications. Moreover, when this study was designed (around 2016), glucose‐lowering strategies emphasizing cardiovascular benefits had not yet been firmly incorporated into clinical guidelines. Also, patients with a history of ASCVD were excluded from the study, reducing the urgency for using such medications. However, the Simplified module does not preclude the use of such agents. Incorporating GLP‐1RA or SGLT‐2i into maintenance regimens should be actively explored, as these agents not only offer cardiovascular benefits but also effectively maintain glycemic control. These two purposes—cardiovascular protection and glycemic stability—are complementary rather than conflicting.

The evolution of T2DM management represents a paradigm shift driven by evidence‐based medicine. While treatment simplification may appear to be a minor advancement on the surface, it reflects a deeper transformation in our understanding of diabetes care—from passively reacting to worsening glycemia to proactively protecting β‐cell function and pursuing disease reversibility. Future research should examine the applicability of this strategy across diverse populations and cultural contexts, assess the feasibility of different treatment modules, evaluate long‐term outcomes related to complications, and analyze cost‐effectiveness. The ultimate goal of diabetes management remains to minimize the disease's impact on both individual lives and society. Thus, no matter how advanced our research and approaches to diabetes care become, simplicity will always remain a fundamental guiding principle.

## Author Contributions

Yanbing Li conceptualized the study, Liehua Liu performed data analysis, and drafted the manuscript. Yanbing Li contributed by reviewing and revising the manuscript. Both authors approved the final version of the manuscript and contributed significantly, in keeping with the latest guidelines of the International Committee of Medical Journal Editors. Yanbing Li and Liehua Liu serve as guarantors of this work.

## Conflicts of Interest

The authors declare no conflicts of interest.
